# Glycolipid and Hormonal Profiles in Young Men with Early-Onset Androgenetic Alopecia: A meta-analysis

**DOI:** 10.1038/s41598-017-08528-3

**Published:** 2017-08-10

**Authors:** Rossella Cannarella, Sandro La Vignera, Rosita A. Condorelli, Aldo E. Calogero

**Affiliations:** Department of Clinical and Experimental Medicine, University of Catania, Catania, Italy

## Abstract

Hormonal and metabolic abnormalities have been reported in men with early-onset androgenetic alopecia (AGA). Although this has been ascribed to the existence of a male polycystic ovary syndrome (PCOS)-equivalent, data on this topic are inconsistent and this syndrome has not been already acknowledged. To evaluate if, already before the age of 35 years, any difference occurs in the glycolipid and hormonal profiles and in the body weight in men with AGA compared to age-matched controls, we performed a comprehensive meta-analysis of all the available observational case-control studies of literature, using MEDLINE, Google Schoolar and Scopus databases. Among 10596 papers retrieved, seven studies were finally included, enrolling a total of 1009 participants. Our findings demonstrate that young men with AGA have a slightly but significantly worse glycolipid profile compared to controls and a hormonal pattern resembling those of women with PCOS, already before the age of 35 years. Therefore, early-onset AGA might represent a phenotypic sign of the male PCOS-equivalent. The acknowledgement of this syndrome would be of importance to prevent the long-term consequences on health in the affected men. The glycolipid profile and the body weight should be monitored in men with AGA starting from the second decade of life.

## Introduction

The association between alopecia and a higher risk of developing coronary heart disease and cardiovascular risk factors has been reported in both genders from a meta-analytic study^1^. Androgenetic alopecia (AGA) has been suggested as an independent predictor of mortality for type II diabetes mellitus (DM II) and cardiovascular diseases (CVDs)^2^. Early-onset ( < 35 years) AGA is a recurrent feature among the male relatives of women with polycystic ovarian syndrome (PCOS)^3^. Therefore, it has been proposed as a sign of the male PCOS-equivalent^4^. The possible existence of this syndrome is of particular interest, especially for its possible long-term consequences on health. Some observational studies evaluated the hormonal and metabolic profile in men with early-onset AGA, finding a PCOS-like hormonal pattern and metabolic abnormalities^5–10^. Hence, early-onset AGA might be a sign of the male PCOS-equivalent, a complex syndrome with a metabolic background.

This study aims to meta-analyze all the available data to evaluate various established indices of metabolic function, namely insulinaemia, insulin-resistance, body weight, total, HDL and LDL cholesterol and triglycerides in men with early-onset AGA. We also evaluated the hormonal profile (luteinizing hormone [LH], follicle-stimulating hormone [FSH], LH-FSH ratio, dehydroepiandrosterone sulphate [DHEAS], total testosterone, sex hormone binding globulin [SHBG]) of these men.

## Results

Using the above-mentioned search strategy, 10596 papers were retrieved. Among these, 69 were judged pertinent after reading the title and the abstract. Therefore, their full texts were carefully read. The study by Acibucu and colleagues^11^ was excluded since the age of cases and controls was higher than that established in the inclusion criteria of this meta-analysis (36.28 [7.74] *vs*. 35.14 [6.54] years). Other studies were excluded for the same reason^12–16^. Some articles were not included because they were study of association without a control group. The study by Ertas and colleagues^17^ was excluded because the authors subdivided patients with AGA into three different groups according to the AGA degree and, similarly, the results were given according to such groups. We contacted the authors to know if they had the values of the entire cohort of patients with AGA, but we did not receive any answer. Finally, the study by Hirsso and colleagues^18^ was not included since the group of control also consisted of men with I and II degree AGA.

**Figure 1:**
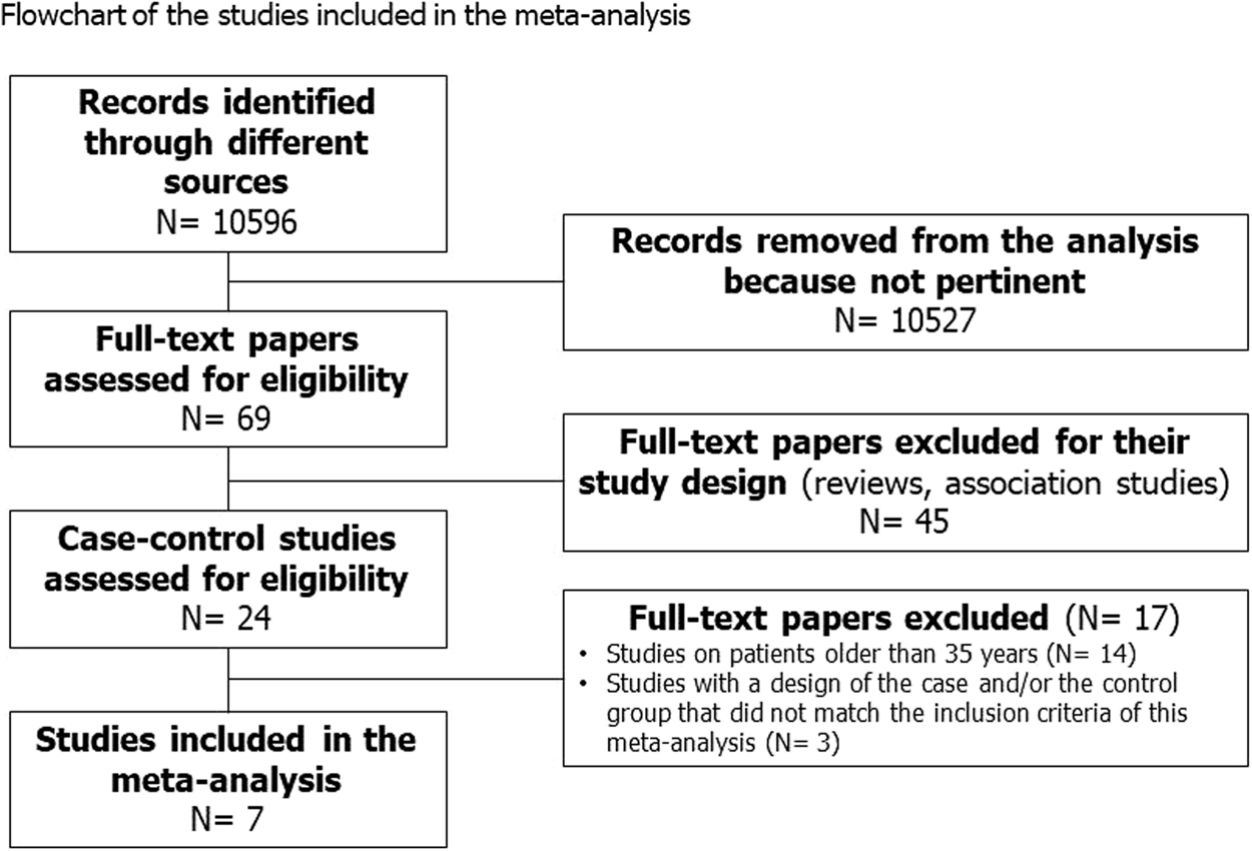
Flowchart of the studies included in the meta-analysis. We identified 10596 papers. 69 were assessed for eligibility. Based on the inclusion criteria of the present study, 7 were finally included in this meta-analysis.

Seven studies^9, 10, 19–23^ met our inclusion criteria and were eligible to be included in this systematic review, with a total of 1009 participants: 522 were men with early-onset AGA and 487 were controls (Fig. 1). However, not all the studies included evaluated the same parameters. A higher number of articles investigating the metabolic profile was found compared to those investigating the hormonal status. We did not considered in the meta-analysis the total testosterone levels of the study by Banger and colleagues^22^ and the SHBG levels of the study by Gonzalez-Gonzalez and colleagues^9^, since they were not comparable with those of the other studies. We unsuccessfully tried to get in touch with the authors.

### Glycemic profile

**Figure 2:**
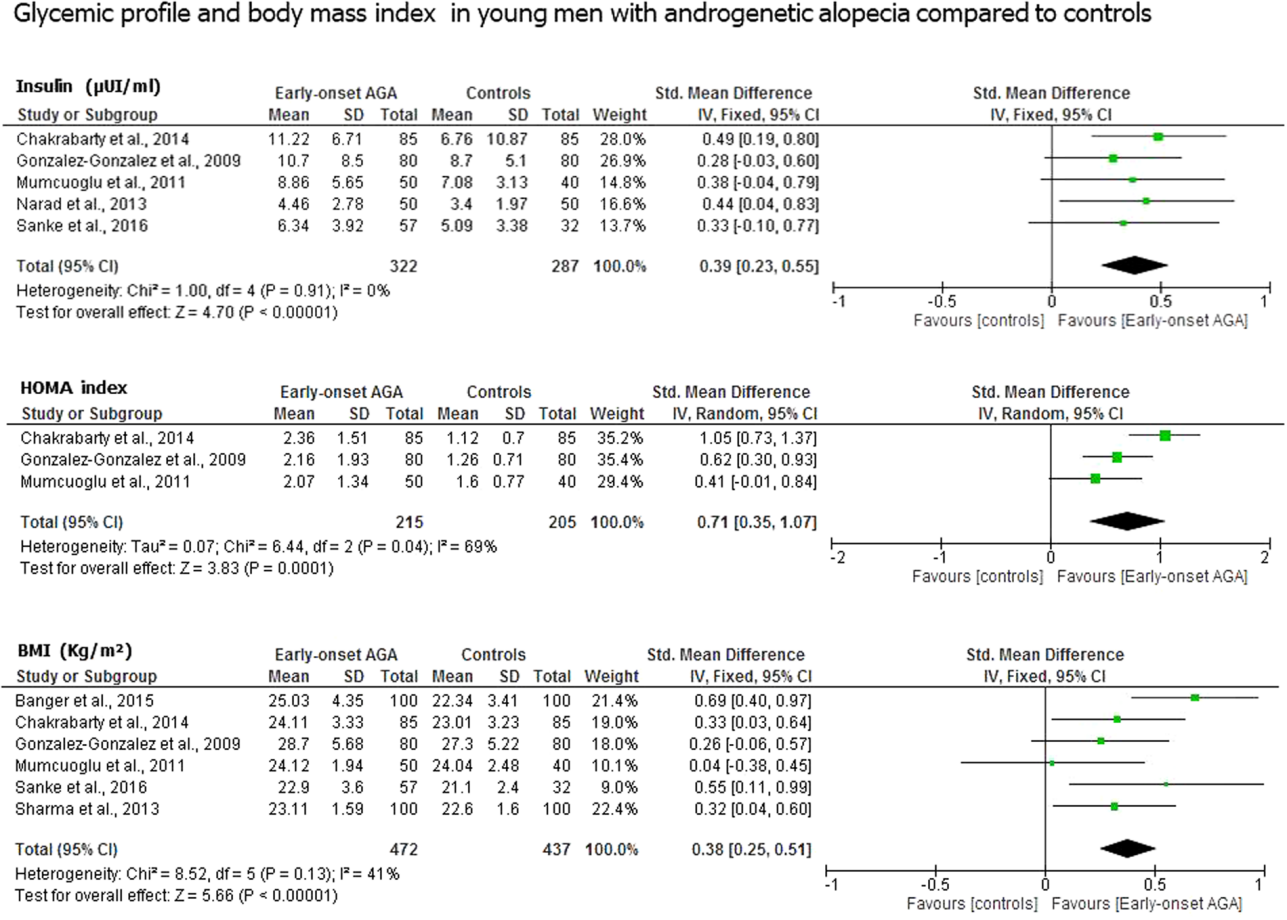
Glycemic profile and body mass index in young men with androgenetic alopecia compared to controls. Men with early-onset androgenetic alopecia younger than thirty-five years old showed significantly higher insulin serum levels, HOMA index, a marker of insulin-resistance, and body mass index values compared to age-matched controls. Insulin was expressed in µUI/ml; Body mass index was expressed in Kg/m^2^.

Insulin serum levels were significantly higher in men with early-onset AGA (n = 322) compared to controls (n = 287) (mean difference [MD] 0.39 µUI/ml; 95% confidential interval [CI] 0.23, 0.55; *P* < 1 × 10^−5^). The HOMA index, a marker of insulin-resistance, was also higher in these men (n = 215) *vs*. controls (n = 205) (MD 0.71; 95% CI 0.35, 1.07; *P* = 1 × 10^−4^) (Fig. 2).

### Lipid profile

**Figure 3:**
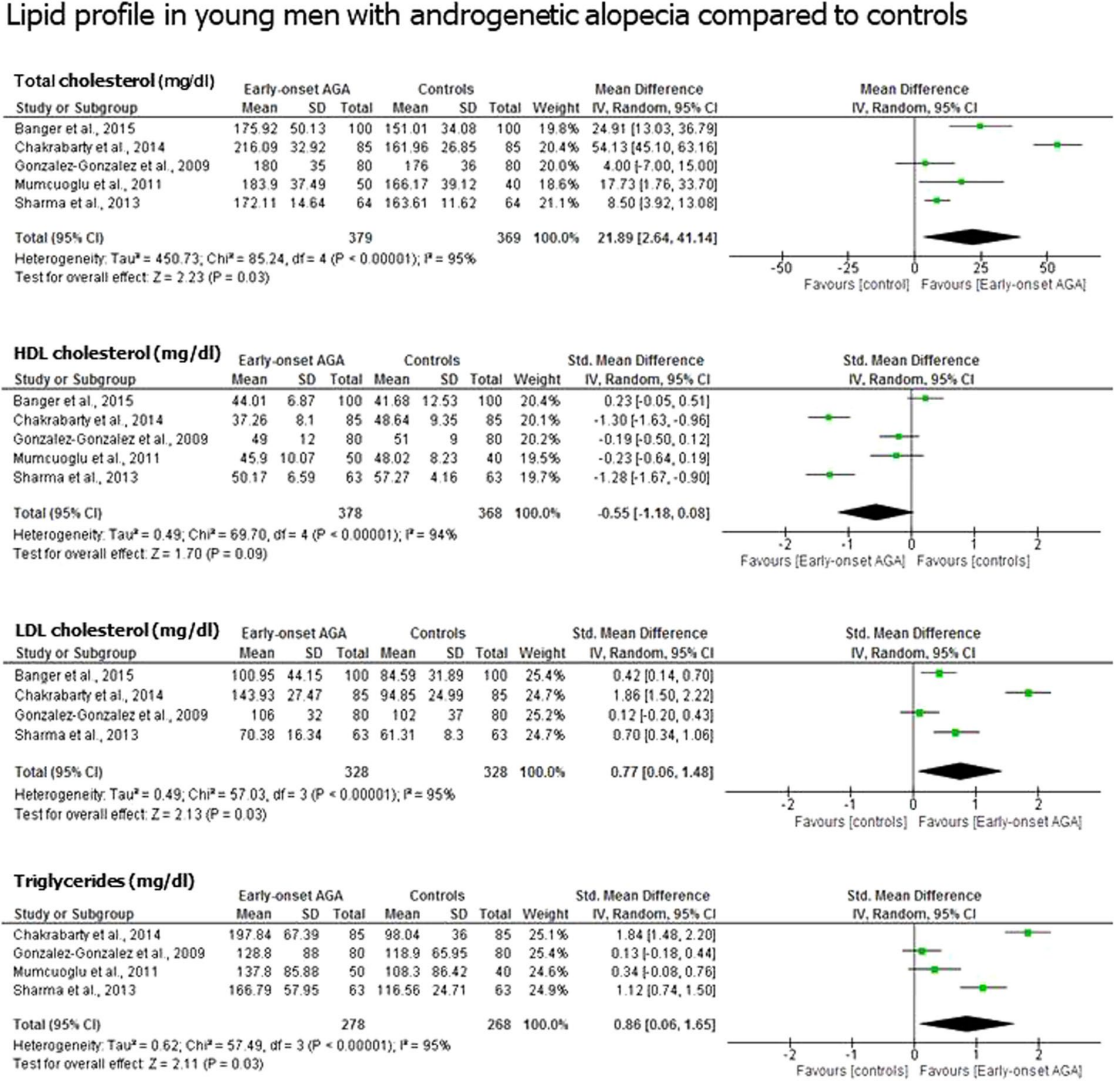
Lipid profile in young men with androgenetic alopecia compared to controls. Men with early-onset androgenetic alopecia younger than thirty-five years old showed significantly higher total cholesterol, LDL cholesterol and triglycerides compared to controls. Also a downward trend for the HDL cholesterol levels was found. Total cholesterol, LDL and HDL cholesterol and triglycerides were expressed in mg/dl.

Serum total cholesterol (MD 21.89 mg/dl; 95% CI 2.64, 41.14; *P* = 0.03) (379 cases *vs*. 369 controls), LDL cholesterol (MD 0.77 mg/dl; 95% CI 0.06, 1.48; *P* = 0.03) (328 cases *vs*. 328 controls) and triglycerides (MD 0.86 mg/dl; 95% CI 0.06, 1.65; *P* = 0.03) (278 cases *vs*. 268 controls) were significantly higher, whereas HDL cholesterol showed a trend towards lower levels (MD −0.55 mg/dl; 95% CI −1.18, 0.08; *P* = 0.09) (378 cases *vs*. 368 controls) in men with early-onset AGA compared to controls (Fig. 3).

### Anthropometric characteristics

Men with early-onset AGA (n = 472) had higher body mass index (BMI) values (MD 0.38 Kg/m^2^; 95% CI 0.25, 0.51; *P* < 1 × 10^−5^) compared to controls (n = 437) (Fig. 2).

### Hormonal profile

**Figure 4:**
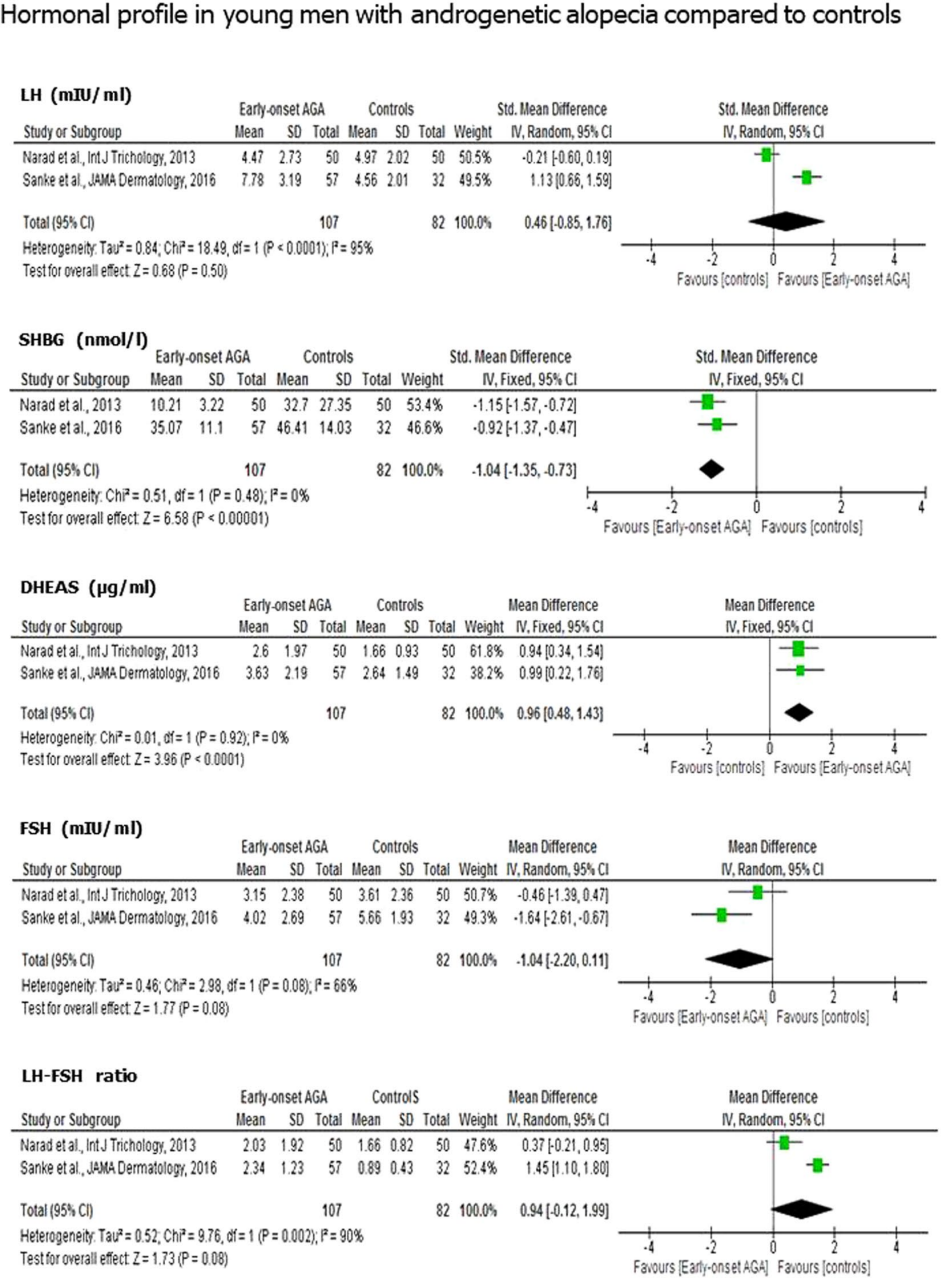
Hormonal profile in young men with androgenetic alopecia compared to controls. Men with early-onset androgenetic alopecia younger than thirty-five years old showed significantly higher luteinizing hormone (LH) and dehydroepiandrosterone sulphate (DHEAS), and lower sex hormone binding globulin (SHBG) serum levels compared to controls. A downward trend for serum follicle-stimulating hormone (FSH) and an upward trend for the LH/FSH ratio was found. No difference in testosterone levels was observed among the two groups. Both LH and FSH were expressed in mIU/ml, DHEAS in µg/ml, SHBG in nmol/l, total testosterone in ng/ml.

Only two studies^19, 23^ evaluated the hormonal profile. Hence, the number of patients and controls meta-analyzed was smaller and comprehended only 107 cases and 82 controls. The results showed higher levels of LH (MD 0.46 mIU/ml; 95% CI −0.85, 1.76; *P* = 0.05), lower levels of SHBG (MD −1.04 nmol/l; 95% CI −1.35, −0.73; *P* < 1 × 10^−5^) and higher levels of DHEAS (MD 0.96 µg/ml; 95% CI 0.48, 1.43; *P* < 1 × 10^−4^) in men with early-onset AGA compared with controls. In addition, we found a downward trend for serum levels of FSH (MD −1.04mIU/ml; 95% CI −2.20, 0.11; *P* = 0.08) and an upward trend for the LH/FSH ratio values (MD 0.94; 95% CI −0.12, 1.99; *P* = 0.08) (Fig. 4).

## Discussion

The results of this meta-analysis showed the presence of a slightly worse glycemic and lipid profile and of slightly higher BMI values in men with early-onset AGA. Moreover, although on a small number of patients and controls, we also observed significantly higher LH and DHEAS levels, and lower SHBG levels, a downward trend for the FSH values and an upward trend for the LH/FSH ratio. Interestingly, these abnormalities were already present in a group of young ( <35 years old) men.

### Metabolic abnormalities and cardiovascular disease

The results on the glycolipid metabolism are in line with evidence clearly showing an increased risk of metabolic impairment and CVDs in men with early-onset AGA. Several authors have shown a positive association between AGA and metabolic syndrome (MetS)^24–26^. A meta-analysis on a cohort of 4006 men showed a 2-fold higher risk of developing MetS in men with AGA^25^. On the contrary, other authors failed to find this association in cohorts of 116^27^, 1707^28^, 184^29^, and 120^30^ men. However, Hirsso and colleagues described lower insulin-sensitivity in men with AGA^31^. Accordingly, Abicucu and colleagues reported a higher prevalence of insulin-resistance and MetS in men with AGA aged 20–50 years compared to age-matched controls^11^. Similar results have been reported also by other studies^19, 32^.

Male AGA has been associated with hypertension^33, 34^ (odds ratio 2.195^33^), atherosclerosis and higher intima-media thickness^17, 35^, dyslipidemia, ischemic heart disease in men less than 50 years old^34^, and, in a cohort of 424 men younger than 45 years, with coronary heart disease^36^. In addition, data from two large epidemiologic studies, the Framingham study^37^ and the NHANES I Epidemiologic Follow-up study^38^ revealed a positive correlation between AGA and coronary heart disease in men.

In line with these evidences, the results of this meta-analysis support the hypothesis that men with early-onset AGA are at risk for metabolic impairment, having a worse (although still normal) metabolic profile already in the second decade of life compared to controls. Therefore, they should be evaluated for glycemic and lipid profiles and their BMI should be monitored early in life to prevent long-term metabolic and cardiovascular consequences on health.

### Hormonal alterations and male PCOS-equivalent

Although based on a small cohort, the data of this meta-analysis suggest the presence of a hormonal pattern partially resembling that of women with PCOS.

The higher levels of DHEAS found in men with early-onset AGA compared to controls suggests that this hormone might play a role in the development of AGA. In fact, in the hair follicle, DHEAS is converted into molecules having higher androgen activity (testosterone, dihydrotestosterone)^39^. Moreover, these higher DHEAS levels may be involved in the pathogenesis of the observed slightly worse metabolic profile and might represent a biochemical feature of the male PCOS-equivalent. Indeed, obesity is associated with the hyperactivity of the hypothalamic-pituitary-adrenal (HPA) axis in men^40, 41^. The hyperactivity of the adrenal glomerular, fascicular and reticular zones has been hypothesized to play a role in the development of insulin-resistance and obesity^42^. Conversely, free fat acids (FFA) (which obese subjects are exposed to) seem to stimulate the reticular zone of the adrenal cortex. In fact, *in vitro* studies on human cortical adrenal cells showed that the exposure to the oxidized fat acid EXODE increases basal and ACTH-stimulated synthesis of dehydroepiandrosterone (DHEA), while it does not have any effect on basal cortisol release. Moreover, ACTH-stimulated synthesis of cortisol decreases^43^. Accordingly, the *in vivo* exposure to high doses of FFA increases the production of adrenal androgens (DHEA and androstenedione) in humans^44^. Finally, the treatment with pioglitazone, an insulin-sensitizer, showed to decrease ACTH-stimulated serum DHEAS levels in adult female resus monkeys^45^, thus showing the stimulatory effect of insulin-resistance on the reticular cortex function in the animal model.

These evidences suggest that the increase in DHEAS serum levels observed in men with early-onset AGA might play a role in the increase of insulin, HOMA-index, BMI. On the contrary, also obesity and insulin-resistance may be effective in increasing DHEAS levels.

The lower levels of SHBG found in men with early-onset AGA may represent a sign of metabolic imbalance. In fact, low levels of SHBG have been proposed as a marker of insulin-resistance and hyperglycemia/DM II in patients with AGA^13^. Along this line, insulin has shown to inhibit the SHBG synthesis in the liver^46^. These lower levels might represent another biochemical sign of the male PCOS-equivalent.

There is a great amount of evidence showing the association between AGA and metabolic-cardiovascular impairment. The present meta-analytic study demonstrates that a worse (but still normal) glycolipid profile already exist before the age of 35 years in men with AGA compared to controls. This association might be due to the existence of the male PCOS-equivalent. As a matter of facts, although only few studies evaluated the hormone profile in men with early-onset AGA since now, the evidence coming from such researches suggest the presence of a hormonal profile resembling that of women with PCOS in these men.

According to the National Institutes of Health/National Institute of Child Health and Human Disease (NIH/NICHD), the European Society for Human Reproduction and Embryology/American Society for Reproductive Medicine (ESHRE/ASRM) and the Androgen Excess Society (AES), the diagnosis of PCOS requires the presence of clinical and/or biochemical hyperandrogenism in women^47^. Hence, polycystic ovaries are not the hallmark of the syndrome. In addition, like in men with early-onset AGA, metabolic abnormalities are a prominent feature in women with PCOS^48^. The recent evidences support the concept of the hereditability of this syndrome: this is of about the 70% and it is inherited thought an oligogenic mechanism^49^. The genetic background responsible for the susceptibility to the PCOS may be inherited also by men, and, therefore, we speculate that the early-onset AGA could represent a phenotypic sign of the male PCOS-equivalent, a complex syndrome with a metabolic background. This would explain the glycolipid and hormonal profile found in men with early-onset AGA, and also the association between AGA and cardiovascular diseases. The acknowledgement of this syndrome would be of importance to prevent the long term consequences on health of these men.

### Limits of the study

The major limit of this study was the small cohort, especially for the evaluation of the hormonal profile. This was also due to the decision to include only studies on men younger than 35 years. We used this strict cut-off to evaluate if any difference in the glycolipid profile in men with early-onset AGA compared to controls could be already found early in life.

In addition, five among the seven studies evaluated in this meta-analysis did not include BMI-matched controls. This may represent a bias since both glycemic and lipid profiles might primarily relate to the higher BMI found in men with early-onset AGA compared to controls. However, the BMI-matched case-control studies^9, 10^ both reported a significantly higher HOMA-index in men with early-onset AGA compared to controls, thus showing that this finding is independent from BMI.

## Conclusive remarks

The results of this meta-analysis showed the presence of slightly worse glycemic and lipid profiles in men with early-onset AGA. This finding is in line with the strong association between early-onset AGA and MetS or CVDs. Hence, monitoring the glycemic and lipid profiles and the BMI in men with early-onset AGA should be suggested to early detect any potential metabolic impairment.

The mechanism by which early-onset AGA is associated with metabolic and cardiovascular abnormalities is still not known. Based also on the hormonal findings, we hypothesize that these men may display the male PCOS-equivalent. However, the presence of hormonal alterations in these men need to be further investigated.

## Methods

### Sources

This study was performed using the MOOSE Guidelines for Meta-analyses and systematic reviews of observational studies. Data were independently extracted by A.E.C. and R.C. A systematic search was performed though the MEDLINE, Google Scholar, and Scopus databases, from each database inception to April 30, 2017. Our search strategy was based on the following key words: “male androgenetic alopecia”, “male premature baldness”, “insulin”, “insulin-resistance” and “polycystic ovary syndrome male equivalent”. Additional manual searches were made using the reference lists of relevant studies. No language restriction was used for any literature search. The authors were contacted for missing data.

### Study selection

Information on the year of publication, country, continent, gender, study design, mean age of cases and controls was collected. Studies which met the following inclusion criteria were included in the meta-analysis:

1. Design: observational case-control studies on men with (cases) and without (controls) early-onset AGA, evaluated through the Hamilton-Norwood scale^50, 51^.
2. Age of cases and controls lower than 35 years.
3. No treatment with finasteride or any other medication.
4. no major comorbidities (including thyroid, pituitary or adrenal disorders, liver or kidney failure) in both cases and controls.
5. No treatment with weigh loss or insulin sensitizer drugs, glucocorticoids, androgens and antiandrogens in both cases and controls.
6. No other types of alopecia apart from the androgenetic one.

Studies that did not meet these criteria were excluded.

The quality assessment of the studies included in the present meta-analysis was performed with the Ottawa–Newcastle scale, which evaluates three separate domains that refer to selection of study groups, comparability of groups and ascertainment of outcomes^52^. The maximum score is 9. Studies with a score < 5 were considered to have high risk of bias, between 5 and 7 moderate risk, > 7 low risk of bias (Table 1).

**Table 1:** Summary of the studies included and their quality assessment^a^.

Source	Location	No. of AGA Patients	No. of Controls	Patient’s AGA degree^b^	Age (years) of cases and controls	Outcomes	BMI-matched studies	Selection	Comparability	Exposure or Outcome	Risk of bias
Gonzalez-Gonzalez *et al*.^9^	Mexico, single institution	80	80	≥III	25.64 [2.68] 26.49 [3.08]	FBG, insulin, HOMA-index, total-C, HDL-C, LDL-C, triglycerides, free testosterone, SHBG	+	****	**	***	Low
Mumcuoglu *et al*.^10^	Turkey, single institution	50	40	≥III	24.32 [2.68] 23.42 [3.0]	FBG, insulin, HOMA-index, FIRI, total-C, HDL-C, LDL-C, triglycerides	+	***	**	***	Low
Narad *et al*.^19^	India, single institution	50	50	≥III	<30 <30	FBG, Insulin, LH, FSH, LH/FSH ratio, total testosterone, FAI, DHEAS, SHBG	−	****	*	***	Low
Sharma *et al*.^20^	India, single institution	100	100	≥III	28.61 (3.03) 28.45 (3.11)	Blood pressure, FBG, total-C, HDL-C, LDL-C, VLDL, triglycerides, BMI, WC, WHR	−	****	*	***	Low
Chakrabarty *et al*.^21^	India, single institution	85	85	≥I	26.44 [2.64] 25.65 [3.19]	Blood pressure, FBS, HOMA-index, total-C, HDL-C, LDL-C, VLDL, triglycerides, BMI	−	***	*	***	Moderate
Banger *et al*.^22^	India, single institution	100	100	≥I	27.03 [5.36] 26.22 [5.1]	Blood pressure, FBG, total-C, HDL-C, LDL-C, triglycerides, BMI, WC, WHR, CRP, ESR	−	****	*	***	Low
Sanke *et al*.^23^	India, single institution	57	32	≥III	24.7 [2.8] 24.2 [2.6]	FBS, insulin, HOMA-index, BMI, LH, FSH, LH/FSH ratio, total testosterone, FAI, DHEAS, SHBG,	−	****	*	***	Low

Abbreviations: AGA = androgenetic alopecia; BMI = body mass index; CRP = C-reactive protein; DHEAS = dehydroepiandrosterone sulphate; ESR = erythrocyte sedimentation rate; FAI = free androgen index; FBS = fasting blood sugar; FIRI = fasting insulin resistance index; HDL-C = HDL cholesterol; LDL-C = LDL cholesterol; SHBG = sex hormone binding globulin; total-C = total cholesterol; VLDL = very low-density lipoproteins; WC = waist circumference; WHR = waist to hip ratio. Values in square brackets represents the standard deviation, whereas those in round brackets represents the mean difference.

^a^The quality assessment of individual studies was based on the Ottawa–Newcastle scale^52^. Possible scores are 0 to 4 asterisks for selection, 0 to 2 for comparability, 0 to 3 for exposure or outcome. One asterisk indicates the lowest score, 4 asterisks the highest score. Studies with a score < 5 were considered to have high risk of bias, between 5 and 7 moderate risk, > 7 low risk of bias.

^b^The degree of androgenetic alopecia was evaluated though the Hamilton-Norwood Scale^50, 51^.

The Cochran-Q and I^2^ statistics were used for the assessment of statistical heterogeneity. Specifically, statistical heterogeneity was tested using the chi-square test. If I^2^ ≤ 50%, the variation of the studies was considered to be homogenous, the fixed effect model was adopted. If I^2^ > 50%, there was significant heterogeneity between studies, the random effects model was used. All P values ≤ 0.05 were considered statistically significant. The analysis was performed using RevMan software v. 5.3 (Cochrane Collaboration, Oxford, UK). For each outcome, the standard MD with the 95% CI was calculated.
